# Community Studies of Tuberculosis

**Published:** 1917-05

**Authors:** 


### Community Studies of Tuberculosis.

In the study of any disease too much stress cannot be laid on the study of man himself. It is therefore particularly encouraging that during the last few years we, in tuberculosis, have been breaking our bonds and have more and more shown the tendency not to remain satisfied with the mere discovery of the tubercle bacillus in any particular instance. A house or a city block swarming with tubercle bacilli may or may not mean a great deal. But it will not explain all that there is to all cases of tuberculosis in infants, adolescents and the aged that come out of that house and city block. So much we are sure of. And we sometimes wonder whether in and of itself it will explain very much more than the opportunity for infection.

That we are thus breaking our bonds is becoming increasingly plain. For the time being we are supplementing the test tube and the experimental rabbit in something more than a speculative way. Community studies are becoming fashionable. We are now reading of the Michigan Survey, the Framingham Experiment and the Saranac Lake Census. All of these, if done properly, will take money. It is the writer's opinion that the importance of the studies will justify the expediture of any conceivable amount of money; that is, if the investigators approach their problems free from all bias and prejudice, if they appreciate its difficulties in even the smallest and most provincial communities and if their enthusiasm runs more to hard work and capable analysis and less to dogmatic conclusion and roseate anticipation of immediate results. We cannot have too much information. We cannot have too little premature classification and systemization.

The Framingham Experiment is already well under way. At the last meeting of the Board of Directors of the National Association, Dr. Armstrong, the executive officer, reported that all elements of the population of the city were lending their earnest co-operation. Not only is there a large local advisory committee at work on the completion of plans for the methods of attack, but the assistance of the State Health Department, the United States Public Health Service and neighboring institutions has already been enlisted. Local insurance agents and the school physician and nurses have finished a sanitary survey and have entered upon a census of the sick; and it is their hope to have all persons whose health is below par undergo physical examination. These examinations will be made under the auspices of the local medical club which includes all the physicians, who for doubtful cases of tuberculous suspects will have the assistance of consulting specialists. An educational propaganda carried on through many channels is preparing the ground for effective work and making ready the people for the enactment of future measures. There are committees on diagnostic standards, health week and prize essays which lend assistance to the director while the medical club is planning a campaign of instruction which will comprise many lectures and clinics on tuberculosis.

It can easily be seen that besides any new information concerning the etiology and prevention of tuberculosis that would be gained by such measures, the experiment is certain to bring about a definite and lasting awakening among the people as to the real nature and meaning of tuberculosis as a community problem.—*American Review of Tuberculosis.*

